# Researches and Experiments upon the Human Blood

**Published:** 1847-10-01

**Authors:** 


Ricerche ed Esperienze sul Sangue Umano. Del Dottore *Gio. Polli.* Serie I. II. III. ed. IV. (Annali Universali, Vol. 106, 109, 113, 121, ed. 122. 1843—47.)

Researches and Experiments upon the Human Blood. By Dr. *Polli.*

Dr. Polli having been much struck, during his studentship at Pavia several years since, with the perusal of the account of some of Hewson's Experiments upon the Blood, resolved entering himself upon a course of experimental inquiry respecting certain points needing illustration; and he has, from time to time, communicated to the Annali the valuable series of papers detailing their results and the therapeutical conclusions derivable from these, an analysis of which we now propose laying before our readers.

The *First Series* has for its title

### Researches and Experiments upon the Formation of the Buffy Coat of the Blood, and its Symptomatic Value in Disease.

After adverting to the contradictory accounts which were formerly furnished of the appearance of the blood in disease, and the rarity and uncertainty with which practitioners employed these as indications of treatment, he observes that the chemical physicians of our own times have effectually revived attention to the subject, and led to the belief that, at least as much information may be obtained from the inspection of the blood as from post-mortem examinations. He then continues:

"The true pathological anatomy of the blood is its analysis, since we can only divide the fluid by means of re-agents. But every hand is not able to wield such a scalpel as the re-agent. This requires a longer and more severe course of study than is necessary for the acquisition of anatomical knowledge. But, without pretending that every practitioner should know as well how to analyze the blood as to examine a body, it is certainly to be desired that he should be enabled to read, in the physical appearance of the blood he removes from a patient, those constant characters, that more or less clearly reveal its intimate composition, and to which they have been shown to bear a necessary relation. The labours of Prout, Denis, Lecanu, Andral, and Gavarret have revealed to us so many facts that, although the pathological doctrine of the blood is not quite complete, the morbid anatomy of this fluid portion of the organism stands upon an equality with that of its solid tissues."—Vol. 106, p. 76.

1. *On the Influence exerted by the Coagulability of the Blood in the production of the Buffy Coat.*—Every one knows that blood drawn from the arm separates in very different periods of time into clot and serum; and the question here agitated is what effect has its more speedy or tardy coagulation in the induction of the buffy coat. Some experiments of Hewson upon this subject, as we have already stated, attracted the author's attention at an early period; and these, with some observations he had the opportunity of making in the hospital, led him to the opinion that a slow coagulation of the blood was highly favourable to the production of the buffy coat. So many chemists of celebrity however support, and so many oppose this opinion, that he deemed it an excellent occasion for instituting a series of experiments, which, from their number and the care with which they were conducted, would be in a position to secure acquiescence in the results they indicated.

It is obvious, in a question of time of this sort, unless great care be taken in ascertaining the precise period of coagulation, great errors may easily occur. The following is Signor Polli's criterion.

"The blood was caught in a conical glass vessel holding about an ounce, and which when nearly filled was left in a state of complete rest. Watch in hand, the instant of complete coagulation was carefully observed. The criterion of this was the witnessing *a few drops of serum transude at the surface of the consolidated coagulum or between the edge of the crassamentum and the parietes of the recipient.* The point at which the blood ceases to be fluid, and solidifies, so that the recipient may be turned upside down without pouring it out, did not seem to me to be so true a point for determining the existence of complete coagulation, as that derived from the transudation of a few drops of the serum, which is to remain permanently fluid, through some portion of the consolidated mass. This fluid, if collected and placed in a watch-glass, remains constantly fluid, undergoing no manner of coagulation. As long as the serum continues imprisoned within the tissue of the clot, rendered more or less dense by its commencing coagulation, we cannot say that such coagulation has taken place; but when the serum is beginning to separate itself from that which was only maintained fluid by the vital influence, and which has now passed into the solid state, the fact is decided."—Vol. 106, p. 86.

The "fixed point" here indicated is of unquestionable importance, since, without the aid of some such test of complete coagulation, different observers will infer this to have taken place at very different periods. Dr. Polli adds that, it very seldom happens that the serum does not then show itself; but still occasionally the coagulum contracts adhesion with the recipient sufficiently firmly to prevent its passage. By gently moving the edge of the coagulum by means of a feather, or even inclining the vessel on one side, the serum is rendered apparent. Blood so observed has led the author to lay down the three following propositions.

(A.) *That blood forming a buffy coat takes a much longer space of time to coagulate than does blood which forms no such coat.*—The blood examined in 405 cases of disease, was found to have required the mean period of 27 min. 30 sec., when it produced the buffy coat, and but 11 m. 50 s. when no buffy coat presented itself—that is to say, a period much less than a half in the one case than in the other. The time required for blood to coagulate, when this was buffy, was found to be nearly twice as long in the case of men (41 m.) as in that of women (23 m.); and that this last generally takes a notably longer time to coagulate than does that of children (18 m.). The cases were taken as they presented themselves without selection in the hospital.

(B.) *Blood which of itself would not produce the buffy coat, presents this whenever its coagulation can be sufficiently delayed.*—Alkaline solutions, employed not in sufficiently strong proportions to maintain the permanent fluidity of the blood, possess the power of retarding its coagulation; and in twenty experiments here referred to, portions of blood, which otherwise gave no buff, were in this way delayed in their coagulation for some hours with the effect of inducing a buffy coat. In like manner, normal blood may be made to produce this by delaying its coagulation through the prevention of the contact of air by a layer of oil or carbonic acid gas.

(C.) *Blood disposed to produce a buffy coat may be prevented doing so by sufficiently accelerating its coagulation.*—An experiment repeatedly performed by the author conclusively settles this point. Let blood from the arm of a patient suffering from inflammation be caught in two separate vessels, one of which is to be put aside at once, and the contents of the other previously stirred for a minute or two by the finger or a glass rod, or the vessel so shaken as to keep them in motion for that time. It is then to be set aside also. The first portion will complete its coagulation in 25, 30, or more minutes, giving a more or less thick buffy coat, but that which has been agitated will coagulate in ten minutes, presenting no buffiness whatever: so that persons who have been shown the two coagula could hardly credit they were produced from the same blood, being disposed to consider the one a specimen of morbid, the other of normal blood. Other means of accelerating coagulation, such as adding water to the blood as it flows from the vein, or maintaining it at the same temperature as it had held in the body, produce the same result. The same thing may indeed be observed oftentimes naturally as well as artificially. Thus, during a venesection, the first blood abstracted coagulates slowly and buffs, while the latter portion coagulates far more rapidly and without the buffy coat. So, too, if we abstract blood from both arms, one of which has been maintained turgid by the application of the fillet some time before, and which has only been applied to the other just before opening the vein: in the former case the blood coagulates rapidly without buff; in the latter, slowly and with the amount of buff usually observed in the particular disease.

2. *Of the Accidental Circumstances which are capable of modifying the Appearance of the Buffy Coat of the Blood.*—The examination of these may lead us to the explanation of the various apparent exceptions to the law laid down. The circumstances which may give rise to variations in the manifestation of the effects dependent upon the coagulation of the blood may be divided into such as are *intrinsic* and such as are *extrinsic* to the blood. The first are the *density* and *temperature* of the blood when it quits the vein: and the second, embrace the *contact of the atmospheric air*, the *nature of the recipient*, the *temperature of the air*, the *agitation the blood is submitted to*, and the *rapidity with which it flows.*

(1.) *Density of the Blood.*—For the estimation of the density of the blood Dr. Polli employs an areometer which is divided into ten degrees an inch apart, each degree being subdivided into ten parts. The density of the blood, in reference to its buffy or non-buffy state, was examined in 180 instances with the following general results.

"That a *lesser density* accompanies *buffy blood*, as a greater density is associated with *unbuffed blood;* since, taking it generally, the density of the buffed is to that of the unbuffed blood as 5·716 to 6·911. Moreover, the density of the blood of women, taken in general, and without taking the buffy condition into account, is less than that of males as 6·142 to 6·575. The coagulation of the blood, also *considered without reference to the production of buffiness*, likewise takes place more readily in women than in men, requiring for the former a mean time 17 m. 34 s.; for the latter 22 m. 44 s."—Vol. 106. p. 116.

Comparative experiments showed that, while the mean density of buffy blood is more than a degree of the areometer less than that of the unbuffy blood, the density of the serum of the same bloods differed only $\frac{4}{10}$ of a degree; so that it may be affirmed that, "the influence of the density of the serum in the production of the buffy coat is very feeble, the principal modification in the characters of the blood depending upon its coagulation being chiefly due to the different density of the coagulable components of the blood, or to the quantity of fibrine held in the fluid state during the interval which precedes coagulation."

Some important practical results may also be deduced from these experiments upon the density of the blood. Thus: *successive abstractions of blood exert an extraordinary influence in diminishing its density.* From two tables which are given, we deduce that successive depletion will reduce the density of the blood more than one half; viz. from 7°·5 to 3°·5, while that of the serum is reduced only from 4° to 2°·5; whence the corollary, that "*bleeding may be considered as exerting a far more evacuant effect upon the coagulable mass held in a fluid state in blood just drawn*, i. e. *the fibrine, than upon the materials held dissolved in the serum even after the coagulation*, i. e. *the albumen.*" A phenomenon of familiar observation, viz. that the blood at the commencement of inflammatory diseases may present little or no buff, but that in future bleedings this may become highly developed—is explained by the *diminution of the density of the fluid by successive venesections.* The effect may not only be observed in successive bleedings, but *at different periods of the same bleeding*, by examining the respective density of portions of the fluid at the beginning and end of a bleeding. Taking the mean of 40 experiments, the first portion possessed a density of 6·127 and the last of 5·96.

Another series of experiments clearly proved that the blood is rendered more dense by inducing an artificial stagnation or engorgement by means of the fillet for some time previously to opening the vein. "*Venous blood then, when it is prevented by any obstacle from circulating freely in its*

*veins, becomes more dense,* or loses a certain portion of its watery principle. Does not this fact constitute a principle pivot of the doctrine which explains œdemas, cellular dropsies, and other infiltrations, by considering them as exudations induced by obstructed circulation ?"

The density of the blood varies as regards *sex* and *age*. It is greater in the blood of men than in that of women, a mean of 90 examinations of each giving 6·437 to 6·170. In adults the density is generally greater than in children. In *Summer*, also, it is more dense than in Spring and Winter, owing to the more abundant transpiration which then takes place. The author has also many times observed a sudden and unusual increase in the density of the blood of certain patients who have been subjected to profuse sweatings or abundant evacuations,

(2). *Temperature.*—The coagulation of the blood being one of the first of the metamorphoses prior to decomposition which the blood undergoes, heat tends to accelerate and cold to postpone it.

"This fact, together with the no less important one of the lesser density of the blood during spring-time, explains to a certain extent the frequency and abundance with which blood becomes buffed at that season; for, without considering the severity of the visceral phlegmasiæ which prevail in the Spring as compared with the Summer, *the slow coagulation of the blood* favoured by the colder temperature and by its own lesser density, may, at the very least, *cause an exaggeration in the appearance of the buffiness,* which merits the attention of the physician, so that he may be enabled to assign their just value to the symptomatic phenomena furnished by the blood in disease."—Vol. 106, p. 137.

(3). *Contact of the Atmospheric Air.*—A number of ingenious experiments are related in proof, that the contact of the atmosphere favours the coagulation of the blood, just as whatever diminishes or prevents such contact retards this phenomenon. Dr. P. objects to the experiments and conclusions of Magendie, Magnus, and others, upon the influence of various gases in inducing coagulation, inasmuch as the mode of conducting the former do not allow of faith being attached to the latter. He describes at some length the precautions he himself took to secure more exact results, and concludes that *nitrogen* and *oxygen,* separately examined, do not seem to promote the coagulation of the blood more than does the air itself; indeed, if there is any slight difference, they seem to retard it: and that *carbonic acid gas* evidently retards the coagulation, so as to induce the formation of the buffy coat more easily than in the normal state. The oxygen, as well as the azote and atmospheric air, after having reacted on the blood which coagulated under their influence, always presented themselves loaded with carbonic acid gas, proceeding doubtless from the blood in which it had been held dissolved.

"*The blood, therefore, coagulates more readily when in contact with the air and its gases than when deprived of their influence, because it yields to them more readily its carbonic acid gas: and it coagulates more tardily in carbonic acid gas, because, besides not being able to emit that which it contains, it dissolves another portion of this gas, which it cannot be doubted has the effect of still longer maintaining it in the fluid state.*"

(4). *Nature of the Recipient.*—Comparative experiments have shown that coagulation takes place more readily in glass vessels than in tin, and much later in leaden ones. The size of the vessels, and the contact of other solid bodies, likewise exert great influence.

(5). *Agitation.*—This, if carried to any extent, as by stirring the blood with a bundle of rods, will prevent its coagulation by depriving it of all its fibrine: but if the blood be stirred or shaken for a few minutes, and then left at rest, coagulation is only hastened. The different capacity of the vessel, or rather the different amount of the mass of the blood collected together, has, as well as the different mode it flows from the arm, great influence in determining the buffy appearance. A series of experiments showed that a small portion of blood was always longer in coagulating and more often buffy, while a large quantity coagulated quickly without buff; and this circumstance is explained by the amount of agitation kept up during the longer time the larger portion takes to flow.

" So that the mere circumstance of collecting much blood in one recipient, for the reason stated, is the cause of its coagulating more quickly, and hence hindering the appearance of buffiness which a small portion would have afforded. A quantity of blood drawn at a time may likewise mask in another manner the natural aspect of the blood. For the last portion drawn possessing a very different coagulability to the first, and coagulating generally much quicker, covers with a stratum possessed of little or no buffiness the lower strata, which of themselves would furnish much more buff, and this imparts to the blood a mendacious appearance. These facts putting us on our guard respecting the absence or slight quantity of buff in blood received during a copious venesection into only one vessel, show us the necessity *of always receiving a small portion separately when we are desirous of drawing any indication from its appearance with confidence.*" —Vol. 106, p. 160.

3. " *Of the Causes of the Coagulation of the Blood, and of its slow or rapid Coagulation in Diseases.*

" From the preceding observations we may infer that the appearance or non-appearance of buffiness of the blood is a phenomenon chiefly dependent upon and expressed by the time employed in its coagulation, notwithstanding that some other circumstances may secondarily induce certain modifications; and the object is now to inquire what is the cause of the ready or tardy coagulation of the blood when removed from the living economy, as this alone gives value to the presence or absence of the buff, when we wish to draw conclusions from the aspect of the blood in disease."

All circumstances which tend to increase physiologically or morbidly the activity of the animal functions lead to a slower coagulation of the fibrine of blood removed from the body; and all the circumstances producing a diminution of vital power lead to its more ready consolidation. This is seen in the more ready coagulation of the blood of women than that of men—the diminution of the power of the fibrine to hold itself in the fluid state in proportion to the amount of blood taken, and to the lateness of the period of the bleeding during which it is examined—the prompt coagulation which takes place on the induction of syncope and of general or partial obstruction of the circulation—and the greater readiness with which it may be induced in the blood of small and weak animals than in large and strong ones. Whence we may conclude—

" 1. That the consolidation of blood drawn from a living animal is a phenomenon which announces its want of vitality, and is the expression of its passage from the condition of a living to that of a dead body. 2. As the longer maintenance of the fluid state of the blood always coincides with the circumstances of the energetic vitality of the animal as represented by the masculine sex, the free action of the vessels, robust constitution, phlogistic excitement, &c.; and its ready coagulation with circumstances of weakness from sex, age, repeated depletion, exhaustion of the nervous power, &c.: so the *time during which the blood remains fluid may be taken as a measure of the remaining vital activity, and the celerity with which it coagulates as the expression of its feeble resistance to decomposition.*"—Vol. 106, p. 261.

Signor Polli enters into some interesting speculations, into which we cannot follow him, upon the amount of vitality remaining in the drawn blood preventing its coagulation, and the nature of this last process, which he regards as resulting from a species of *spontaneous crystallization* of the fibrine held by the blood in solution. After examining into various opinions upon the *causes of the coagulation,* he thus states the result of his own investigations upon the effect of *carbonic acid* in inducing it.

"1. That although the blood owes its primary tendency to coagulation to a cause as yet unknown, the variable quantity of carbonic acid gas existing in it seems the cause of its variable coagulability in different physiological or morbid circumstances, and therefore one of the principal agents in determining buffiness. 2. That in respect to the symptomatic value of the buff, it may be laid down that a light buffy coat, without frothiness, placed upon a coagulum of very deep colour, not only indicates a slow coagulation of the blood, but also the accumulation of a large quantity of carbonic acid gas in it; while when a rose or scarlet coloured layer, almost always frothy, presents itself upon a not very black coagulum, it announces that such blood is loaded with a large quantity of oxygen." P. 285.

4. The *Fourth Section* being occupied in a critical examination of the opinions of Andral, Gavarret, Giaccomini, and Mandl, upon the formation of the buffy coat, we pass it over, as the space we have at command barely suffices for the exposition of the views entertained by our author himself.

5. *On the Signification of the various Appearances of the Coagulum in relation to its Density, Coagulability, and the Proportion of its Component Parts.*

The examination of blood 12 or 24 hours after its extraction may furnish valuable indications to the physician who may not have had the opportunity of observing it at an earlier period.

"The blood is *plethoric,* or with a prevalence of red globules, when it presents a voluminous crassamentum of a rather bright red colour, soft, with little separation of serum, and which at most lets fall to the bottom of the vessel a certain quantity of separated globules, which form a stratum of rather deeper colour. The abundance of the globules is such, that the fibrine can not only not compress its lamellæ so as to acquire tenacity, but it is unable to comprise the whole of the globules, and lets a portion fall from the coagulum, which by their weight always gravitate to the bottom of the vessel. In this case the blood is never buffy, but is covered with a red foam, or by a border of a reddish colour. But the blood may be plethoric and also present the (so-called) buffy coat, which then has a whitish or greenish white colour, and a gelatinous consistency, the clot being of a deep-red colour, having a layer of coloured globules lying at the bottom of the vessel.

"The blood is *anæmic* and poor in globules when it presents a small clot of a light red colour, mostly hollowed above and swollen out below. It is submerged and sometimes floats in a large quantity of serum, which may be quite transparent, but which is oftener rendered somewhat turbid by a little of the suspended colouring matter. The clot is tenacious and resistent because the fibrine, existing in disproportionate quantities to the other principles, has not its interstices filled with globules, so that its fibres can better approach each other and display a greater power of aggregation. And it is frequently swimming in the serum, because not loaded with globules, it assumes the position which comports with the specific gravity of the fibrine. The serum is reddish from its water dissolving a portion of the colouring matter." P. 305.

Dr. Polli enters at considerable length into the description of the various kinds of fibrinous crusts which are formed upon coagulated blood, and the indications these furnish of slow or rapid coagulation, &c., but we have not space to follow him in this.

### Series II.—On a New Criterion for the Regulation of Bloodletting.

This criterion is deduced from the author's former series of researches, and confirmed by practical application in the Hospital. It is thus stated to be "*The period coagulation of the blood observed at different intervals of time between the abstractions, and in different portions of the mass taken during one blood-letting;*" and is amplified as follows:

"1. Every time a large abstraction of blood is practised, so as to lead to lipothymia, *the last portion* of that removed always coagulates with *greatest promptitude*, whatever may have been the time occupied by the first portion in coagulating. 2. Whenever, on the contrary, upon a person suffering from sanguineous congestion of the nervous centres, asphyxia, apoplexy, &c., bleeding is practised, and, by its use, the vital functions are again set at liberty, the last portion of the blood so removed coagulates *much more slowly* than that which was first emitted. 3. That it suffices to interrupt in some manner the course of the blood in the vein, or to diminish, by means of a ligature applied to an extremity, the irradiation of the nervous power, in order to secure the speedy coagulation of blood, which, a short time after, owing to the obstacles being removed, may re-acquire the power of remaining long without coagulation, 4. That in diseases decidedly of an inflammatory and grave character, during which for the safety of the patient repeated bloodletting is requisite, if, on the occasion of every venesection, the coagulation of the first and last portions drawn be examined; it will be found that at the beginning the coagulation of the latter portion takes place *subsequently* to the former, and continues to do so in an equal ratio to the development of the morbid process, until this reaches its height. From this point, however, as the disease commences declining, the coagulation of the blood of the latter portions *precedes* that of the former. 5. That in the cases in which abstraction of blood has been desisted from for some days, when the *slow coagulation of the last portion* taken announced a continuance of the phlogistic increment and the tolerance of bloodletting, it has become necessary to resort anew to this therapeutical agent, which can in no case be laid aside with the ready cure of the patient, unless the latter portion of the blood manifests an opposite disposition to that now pointed out. 6. That in opposite cases in which the abstraction of blood is persisted in, *notwithstanding its rapid coagulation* after all the venesections and during the two extreme periods of the same one, it has to be speedily renounced in consequence of the symptoms of intolerance which manifest themselves ; and in those few unfortunate cases in which bloodletting is obstinately persevered in under the guidance of fallacious symptoms, vital exhaustion cuts short the career of the patient much more rapidly than would have done the course of the disease.

"It results then from these observations that the maintenance of the fluid state of the blood, comparing one bleeding with another, or different periods of the same bleeding, is a measure of the vital energy proper to the individual, and of that brought into play by the morbid process; and that hence may be determined tolerance and indication of blood-letting ; as on the other hand a prompt coagulation of the blood announces diminution of vital energy, or its exhaustion by the pathological action; and in every case that the power governing the phlogistic or morbid vital movements is lowered." Vol. 109, p. 65.

The criterion is of easy application, the first and last portions of blood drawn being separately collected in glass vessels, and placed at rest beyond the influence of disturbing causes before adverted to. As the difference of time employed by the blood in coagulating depends both upon the condition of the individual and the amount of blood drawn, the criterion in question may not only serve as a guide in judging of the propriety of bleeding in a certain contingency, but may determine the exact quantity to be drawn, and the period of its repetition.

"Let an individual be bled to faintness, and you will always have the last portion of the blood rapidly coagulated, and consequently deprived of buffiness. Receive the blood into six, eight, or ten small recipients, of a similar form and nature, and the coagulation in the first will be in exact relation with the disposition of the fibrine to maintain itself in the liquid form proportionately to the particular physiological or morbid state of the organism; while in the last, such disposition will become gradually paralysed and almost destroyed from the gradually increasing effect of the abstraction itself. By contemplating this phenomenon, which is always a result bearing proportion to the two influences above alluded to, we are enabled to lay down a rule for in some cases practising abundant blood-letting at one time, in others practising it at intervals, or in small quantities; or again simply interrupting its flow once or twice for some minutes during the abstraction, &c.—accordingly as we may be desirous of obtaining a sudden subdual of the morbid exuberance of the vascular activity, or of securing a copious depletion without too great exhaustion of the strength, or the functional disturbance ensuing upon lipothymia, which may injuriously affect the regular course of some affections.

"From the different coagulation of the various portions of blood we may, moreover, as we have said, measure the *intensity of the inflammation* and the *tolerance of the individual ;* or, as others would express it, we may measure the morbid capacity and the amount of diathesis. There may, indeed, be a case in which the first portion of blood drawn indicates by its very slow coagulation a very high pathological condition, while the last portion announces in its rapid coagulation that the emission of blood has completely lowered the powers. This phenomenon may be dependent upon the existence of a very circumscribed, though a very intense affection, or upon exhaustion induced in an individual primarily possessed of very feeble powers of organic re-action; and in such a case bleeding must be most reservedly employed, and frequently entirely rejected, for the reason that it is a lesser danger to leave the disease to proceed unchecked, than to have recourse to means which remove it and the patient together. This difficult pathological circumstance, which a celebrated Italian physician justly compares to an *island of fire in a sea of ice*, is already known to practitioners as one which requires in the use of antiphlogistic measures great regard to be paid to the failure of the general strength. But, unfortunately, it has not always been easy to establish its diagnosis in time, or before unadvisably energetic therapeutical procedures have been put into force. But the criterion I propose informs us of these two opposite conditions co-existing in the same individuals, and measures their degree with a facility and security that no method of investigation hitherto recommended in these difficult cases can boast of." Vol 109, p. 70.

To the objection that the criterion only comes into operation after the abstraction of blood, Dr. Polli observes that, in ordinary inflammatory diseases, the repetition of the bloodletting is the point to be enquired into; and that, even in those rarer cases in which the diagnosis is very obscure, and in which a first bleeding might prove the cause of safety or of death, no harm whatever, and much good, would result from a very small *exploratory* venesection, and made in the view of obtaining the desired information. Such, consisting of one or two ounces, received in two separate vessels should be instituted in all obscure cases of this kind, before resorting to an ordinary venesection. "Perhaps even those small bleedings should be practised in all diseases indistinctly as a means of exploring the condition of the blood, for the same reason that, since auscultation has been employed upon all patients, it has not unfrequently revealed latent morbid conditions, to which the attention of the practitioner might otherwise not have been called until a more remote and a too late period." A small subtraction which can do no harm to the economy, will yet depict to us the true characters of the vital condition of the tissues, and of the amount of the exaltation of the vascular activity and nervous function. It often suffices for the discovery of those circumscribed and concealed phlegmasiæ, which, frequently not spreading to such organs as would furnish external symptoms of their existence, pursue their undermining course until they have reduced the viscera they affect to such a condition, that some acute contingency at last suddenly betrays their formidable character.

"Although the preservation of its fluidity by the blood, or the more or less time it requires for coagulation, constitutes for us the most certain measure of the activity of the phlogistic force, this is, however, only durable in its indications in proportion to the stability of the morbid process itself. The phlogosis may, during its course, spontaneously increase or diminish in intensity, accordingly as it extends to neighbouring tissues, or is confined to those first invaded. So that the different resistence of the blood to coagulation which in every case announces with a rare exactitude the present state, cannot be extended, except within certain limits, to the indication of that which is to follow; since this latter can only be the complex and simultaneous effect of the condition of development of the pathological lesion, and of the modification which the blood-letting itself may have induced. Our criterion, as expressing the present state of the organism, and the impression which the bleeding has developed, furnishes indications which are available for about twelve hours after, and may continue to be so for a much longer period, even to the supervention of complete health, providing new morbid causes and accidental inflammations do not supervene and complicate the course of the disease. And of this we may assure ourselves by the repeated observation of the coagulation of blood taken at brief intervals; since the times employed in the coagulation of the blood taken at the successive abstractions will generally glide, whether these are diminishing or increasing, gradually into each other, sudden variations not being observable save when exacerbations or irregular complications coincide." P. 73.

In corroboration of the above views tables are furnished of twenty cases of inflammatory disease observed in the hospital, for the relief of which were collectively performed 147 venesections. Notes to each case reported exhibit the author's views of the amount of corroboration derivable from it. Some of these are highly interesting, but we have only space to notice some of the practical conclusions he arrives at.

"The observations already made upon the indications the physician may draw from the observation of the coagulation of the blood, and the clinical cases adduced in confirmation and illustration of this criterion, clearly prove that its value rather lies in its enabling us to fix a limit to the abstraction than in encouraging its continuation. And, in fact, if we are not deceived, the comparison of the coagulation of the first and last portions may, independently of the presence of all other symptoms, distinctly indicate whether the evacuation will tend to normalize the vital powers of the functions of the organism, at one time liberating them from oppressive congestions, and at another from the obstacles presented by the excessive and unbalanced action of the nerves, or whether it attacks them with all its impoverishing effects, and directly exhausts the forces necessary to the carrying on of life. Of the two indications which this sign offers the last is not only the most important, since its neglect almost amounts to a fatal result in the disease, but it is also the most attainable, or at least the best supported by facts. The cases referred to show that, if the coagulation takes place with a certain celerity, and this manifests itself repeatedly, and goes on increasing with the bloodletting, we cannot persist in the measure without losing the patient; while the patient hardly ever dies when it is suspended prior to the coagulation having acquired great rapidity.

"*It is not necessary for the complete cure of an inflammation to contiuue the bleedings until the blood no longer gives any buffiness; while it is absolutely necessary to cease the emission when the blood coagulates more rapidly than in the normal state.* The production of buffiness of blood of equal coagulability, as shown in the former series, is always rendered more easy and in larger quantity after a certain number of bleedings than at first, in consequence of the diminished density which the blood acquires; which naturally always much diminishes the phlogistic expression and the consequent indication for bleeding drawn from the crust that covers the blood after a certain number of emissions. The crust or buffiness, in fact, not being *essentially* produced by an increase of fibrine, by a diminution of red globules, or by an attenuation of the serum, but arising from a certain slowness of the coagulation—(of that faculty by which, in certain morbid conditions of the organism, and especially under the influence of the phlogistic process, the fibrine has acquired the power of maintaining itself in a state of fluidity for a period always much longer than in the normal state), it may at once disappear by the operation of whatever modifies that slowness. When the phlegmasia is subdued, and the morbid reactions give way to healthy movements the blood will cease to contain fibrine in a hypersthenic condition, and will then undergo coagulation in a period of time that does not permit the appearance of the buffiness. It happens not infrequently that if, for some reason independently of a reproduction of the phlegmasia, we draw blood during the advanced convalescence of a severe inflammation, in the treatment of which bleeding had been suspended, while the blood was yet covered with a firm phlogistic crust, it will now be found to present no trace whatever of this. A patient may have blood in circulation which if drawn would furnish a buffy crust, and who will yet be perfectly cured without blood-letting. This change in the blood within the vessels, without profuse crises inducing the belief that the morbid matters supposed to be indicated by the buffiness had been evacuated by other channels, frequently excited the surprise of the ancients; but, faithful to observation they had nevertheless laid down as a canon, *Ob solam crustam inflammatoriam venæsectio repetenda non est.*' (*Quarin Met. med, Inflam.* p. 70.)" Vol. 109, p. 104.

Series III.—On the Condition of the Fibrine of the Blood in Inflammation.

A question touched upon in the last extract quoted is treated more at length in the present series of observations :

"The fibrine of the blood exists in a perfectly fluid state in the blood while within the vessels of a living animal, and for some time after being drawn from these. This fluidity, however, is not like an ordinary solution of a solid body in a liquid menstruum. It is true that the alkaline salts hold the fibrine a long time dissolved, out of the vessels, and will re-dissolve it if already coagulated; carbonic acid, too, retards the coagulation to such an extent that it also may be termed a solvent of the fibrine; but the fundamental reason why fibrine is in a fluid state within the vessels of a living animal, and why out of these it more or less readily consolidates, is entirely independent of the alcalescence of the serum and the impregnation with carbonic acid. It is intimately connected with the peculiar vital formation of the tissues, among which a few minutes before it had been circulating. Negative electricity seems also to retard coagulation, but it is not yet clearly made out whether the influence it exerts is purely dynamic, or whether this does not rather arise from the chemical changes induced in the solutions of the salts which are found in the serum in contact with the fibrine. The fluidity of the fibrine in the blood is a species of *vital* fluidity, and its *coagulation* is a cessation of this vital property. Although chemists may demonstrate fibrine and albumen to have the same atomic composition, out of the animal body, and from their isomeric characters, may assume each substance will be similarly effected even within the body by chemical influences—and thus simplify the chemical history of those two substances—they will not by that have removed the necessity of their physiological distinction. When we see them in the laboratory we may allow that they are the same organic material the fundamental composition of which is termed by the moderns *protein*, and it is a matter of indifference whether the same name be given to the two or not: but when we observe them in the living animal, or just extracted from its vessels and yet endued with vital characters, we then see the necessity of distinguishing them by distinctive appellations. One of these materials (*the albumen*) remains permanently fluid even when out of the vessels, after the coagulation and even the destruction of the clot; the other (*the fibrine*) more or less readily loses its fluidity and solidifies. Such a difference is amply sufficient to prove the necessity of not confounding together these two organic materials whatever may be the identity of the remote principles which analysis discovers in them. The names *fibrine* and *albumen* then, should be preserved at least by physicians, as signs expressive of the two physiological characters peculiar to the mode of existence of this organic material during its functions in the vital circle; while when they are divested of the characters they owe to vitality their radical composition will be most conveniently expressed by the chemists by the term *protein*."—Vol. 113, p. 333.

Inflammation may modify the fibrine of the blood in three ways: by increasing its quantity; retarding its coagulation; and by rarefying it.

1. *Increase in the Quantity of Fibrine.*—The author's investigations by means of the areometer confirm the conclusions arrived at by Andral, Becquerel, and others, that in inflammation there is a marked increase of fibrine; and he believes that from the researches of Becquerel, Rodier, and Mulder, it may be deduced, "*that this increase of fibrine is due to the oxidation of a proportionate quantity of the albumen of the serum of the blood.*"

2. *Delay in the Coagulation of the Fibrine* (*Bradifibrina*).—Healthy fibrine ordinarily coagulates in a few minutes after the blood has been drawn. If the individual furnishing it is very enfeebled, aged, or very young, oppressed with plethora or overcome by faintness, the coagulation takes place so rapidly as even to occur around the aperture of the vein whence it flows. On the contrary, when the person is suffering from inflammation, it mostly coagulates slowly enough to allow of the formation of the buffy coat, and sometimes so great is its resistance to coagulation, that it may remain fluid for several days first.

An interesting example is cited, in which the blood first drawn from a patient suffering under severe pneumonia did not coagulate for fifteen days, when it formed a coriaceous crust. This man was bled ten times, and after each bleeding, just as the inflammation shewed more disposition to yield, so did the blood coagulate more readily, and after the last venesections it did so in a few hours. Many of the cases of dissolved and incoagulable blood related by various authors have been mere examples of slow coagulation, in consequence of the persistence of the morbid process; and ignorance of this fact has led to the dangerous practice being so frequently followed of taking the indication for repetition of the bleeding from the ready coagulation of the blood, and the contra-indication from the persistence of its fluid condition!

"A question here at once arises. In what light are we to regard all the descriptions of non-coagulable blood consigned by authors in their histories of putrid and malignant diseases? Are we to reject them as false or admit them as characterizing diseases which are no longer met with? We incline to the belief that the excellent authors who have spoken of dissolved blood, and of blood which would not coagulate, have for the most part badly, or at least incompletely, observed, and have been induced by the condition of their own mind, or the influence of some dominant theory, too hastily to conjoin certain properties with certain physical appearances. And that such an error may have easily and frequently occurred, and will do so again, for a longer period than we could desire, the case above referred to suffices to prove. The blood at the first bleeding possessed all the characteristics by which the older physicians were accustomed to judge of it as dissolved and incoagulable, and indeed a practitioner of our own times, unless his attention had been called to the subject, would not have probably given a different opinion. What would be said of blood which had continued fluid for more than a week, and presented such an absence of the so-called plasticity as to allow of its being mixed together like so much serum, and which deposited at the bottom of the vessel a mass of deep coloured red globules destitute of all cohesion? Would it not be said that this was the dissolved blood of an adynamia, awanting in fibrinous principle, or tending to putrefactive dissolution? Well! This same blood, in process of time, presented the firmest crassamentum, and a most decided buffiness. So far, too, from furnishing signs of putrefactive decomposition, it did not exhibit these for a month, while an equal quantity of blood taken for an ordinary disease of the chest, and exposed side-by-side with it to the same air and temperature putrefied in a fortnight. The individual, likewise, who furnished this blood, so far from suffering under adynamia or any cachexia, was the prey of a violent inflammation which the most active curative measures, consisting of twelve bleedings of 12 ounces each practised in eight days, &c. &c. hardly sufficed to subdue; and nevertheless the cure took place after a very short convalescence, so that 20 days after he had been received at the hospital he was enabled to leave it again in full health.

"For a long time past I have used every diligence in our large hospital (at Milan) to discover some examples of this dissolved, non-coagulable, blood, as described by some pathologists; but I have not been able to find one solitary case, in which blood, *left to itself for a sufficient time,* and protected from external destructive influences, has not undergone a distinct coagulation, prior to putrefaction. Many of those I examined had remained for days in a fluid state, and many had all the appearances justifying completely the prediction of incoagulability, resolution, &c.: but with some care I was always enabled to demonstrate the coagulability. More than once also it happened to me to see blood coagulate which had been taken from the veins of a body 36 or 48 hours after death. And in respect to this, I may observe in passing, that it seems to me that the conditions of rigor or relaxation of the parts of a dead body are in every case dependent upon the state of coagulation of the fibrine in its capillaries, whether this is accomplished, retarded, or re-dissolved."—Vol. 113, p. 348.

The author details at some length the particulars of five of the cases observed by him in the hospital, and a hasty examination of which would have led to their being set down as examples of the dissolved state of the blood. He concludes by repeating his opinion that the *coagulation of the blood prior to its putrefaction is in all cases inevitable;* that the blood in scorbutus and typhus which authors have noted as exemplifying its dissolved condition, is blood more or less poor in fibrine, and more or less slowly coagulable; but that, according to the quantity and quality of the fibrine, it does coagulate sooner or later, and those who have not seen its coagulation and yet noted its putrefaction, have observed it too early or to late. For the expression of long-delayed coagulation he proposes the term *Bradifibrina* (*βραδυς, slow*), as merely indicating slowness, without implying any opinion as to the cause of this.

4. *Rarefaction of the Fibrine* (*Parafibrina*). From various experiments which he undertook the author concludes "that the fibrine of the blood may, under morbid circumstances, assume a lesser density, or become rarified; that fibrine so modified imparts its tenuity to the mass of the blood in which it exists; and that in such condition it at the least is of lesser density than is the albumen of the serum." This, however, does not hold good of all the fibrine of the blood contained in an organism suffering under inflammation, but probably can only be extended to all that of new formation, or which is directly produced by the inflammatory process itself. The blood in the normal state holds fibrine in solution, which by coagulating, renders the liquid residuum of a lesser density, as is the case with every solid body which had previously been dissolved in a liquid of lesser specific gravity. The fibrine rendered slow in coagulating by inflammation, *bradifibrina,* also renders the fluid more dense in which it is dissolved, and lessens its specific gravity upon separating from it. "But there is a third species of fibrine, which is the modified fibrine upon which we are now especially treating, and which has the peculiar property of rendering the liquid in which it is found specifically lighter. We propose to term it *Parafibrina,* in imitation of the chemists who thus distinguish isomeric substances, or those which, preserving the same composition, are yet modified in their properties."

*Parafibrine* in general coagulates with exceeding slowness, so as even to surpass bradifibrine in that respect. Its coagulation, too, takes place in such slender filaments as to be hardly recognisable by the naked eye, and giving rise, together with the enclosed serum, to a mass possessing rather a gelatinous than a fibrous appearance. The delicate meshes thus formed may be to some extent compared to the cellular network which gives something like consistence to the albumen of the white of an egg, or to that which supports the vitreous humour of the eye; but, when separated by pressure from the serum it contains, it is found to be a fibrous tenacious body. The serum raised under the epidermis by blisters or slight scalds is especially rich in parafibrine, and its properties can be best studied in this fluid. When parafibrine coexists with ordinary fibrine or bradifibrine, it coagulates at a later period upon the surface of this as a gelatinous deposit of varying extent: and blood which retains its fluidity for a very long time always contains it. "There are cases in which the blood presents very little crust, although the crassamentum possesses great consistency, and is covered with a thin stratum of a tremulous gelatiniform buff. The production of parafibrine in such cases does not seem to be accompanied by a proportionate augmented quantity of ordinary fibrine, or with the production of bradifibrine. The three modifications of fibrine now noticed may exist independently of each other in a given specimen of blood, and in very different quantities; just as they may all three exist in the same blood, likewise in different proportions."

Signor Polli adverts to the fact of Hewson and Dr. John Davy having already expressed their opinions, that the production of the buffy coat is in some measure due to the greater tenuity of the fibrine; but infers, from a passage in Mr. Wharton Jones' Report in 1843, that these had received little or no sanction among their own countrymen.

*Applications.*—"The character we have recognised in Parafibrine, of possessing a greater tenuity than the serum in which it is found liquified, furnishes the explanation of a pathological phenomenon of great moment; I mean of the fibrinous or plastic transudation, and the sero-fibrinous transudation which constitute the termination of the greater part of the membranous inflammations. Great surprise has always been excited by the dense strata of fibrine which have been found on membranes which have undergone inflammation, and the large pseudo-membranes or flakes of coagulable lymph which are seen swimming in the serum contained in the cavities of inflamed membranes; and the explanation of these familiar facts has continued a desideratum.

"It certainly was far easier to imagine that extravasated serum became coagulated, than to believe that fibrine, which out of the blood-vessels is the body to which the blood owes its consistency, tenacity, and durability, could pass through the walls of the vessels. Nevertheless, it is demonstrated that not only are these morbid transudations of a truly fibrinous nature, but that they transude through the vessels, and coagulate sooner or later, just as does blood which has been drawn. If we examine these products in the dead body, we certainly only find them in their solid form; but the evidence of practitioners is not wanting to shew that, in the fluid evacuated from the chest during life for a pleurisy, the serum has been found in a short time the consistency of a jelly. So, again, pus evacuated in empyema or abscess has, after a while, separated itself into clot and serum; serum evacuated for ascites supervening on peritonitis, or from a hydrocele consequent upon inflammation of the tunica vaginalis, has been converted by coagulation into a tremulous mass. That kind of gelatiniform crust which often covers the surface of blistered parts is but a fibrinous deposit, which, at the beginning, consisted of but a very fluid exudation."—Vol. 113, p. 367.

The author adds that this lightness and tenuity of the parafibrine furnishes also an explanation of a fact which may sometimes be observed in certain stages of inflammation, and which by some has been adduced as an argument in opposition to the law of the formation of the buff of the blood laid down in the foregoing pages—viz. the case in which a thick buffy coat is found on blood which has coagulated with a certain amount of promptitude. Suppose that in such blood there exists a sufficient amount of parafibrine to rarify the fluid, and that it has been drawn after several prior emissions; being thus greatly diluted and quickly coagulable, the conditions are present which permit the red globules to rapidly descend, leaving on the surface a clear stratum of fibrine, which also, after a little time coagulating, may give a thick buff.

The following are some of Dr. Polli's observations upon the conclusions to be drawn from the different appearances of the fibrine.

"These various modifications of the fibrine seem not only to characterize inflammation in the most distinct manner, but each seems to connect itself with a particular degree of intensity of the inflammatory process. Thus: the simple *increase of the quantity* of the fibrine indicates the development of the first stage of an inflammation, the extension of which will be measured by the proportionate excess of this material. The *retardation of the coagulation* indicates an aggravated degree of the inflammatory process, inducing that modification in the fibrine we have termed *bradifibrina.* Lastly, the highest degree of a phlegmasia gives rise to that modification, by reason of which the blood presents the gelatiniform crust, dependent upon the production of *parafibrine.*

"If the coagulum forms with sufficient promptitude to prevent buffiness, and does not give it even when coagulating somewhat slowly from its great density, but is itself consistent and hard, it is an indication that in such blood there is *simple excess or abundance of fibrine.* The phlogosis is only in its first stage, and it will become more or less extended in proportion as the quantity of fibrine is increased. If there is a clot not only consistent and hard, but likewise furnished with a thick stratum of a white coriaceous crust, this having been formed by a proportionally slow coagulation, this is an indication not only that there is an inflammation of some extent, but that it has reached that higher degree of intensity, in which a *certain quantity* of *bradifibrine* is produced. If, on the contrary, the crassamentum is observed to possess but slight consistency, approaching in that respect to a normal condition, but that it possesses a little of the gelatiniform crust or buff, the inflammation which so modifies the blood has reached its highest degree of intensity, but is circumscribed; while we must regard it as both intense and extensive when this gelatiniform crust of *parafibrine* is abundant, and also accompanied by a greater or less share of bradifibrine. This highest degree of the inflammatory process may exist in some points of a tissue and manifest itself as just stated, while in other portions of the same tissue a milder phlogosis, competent only to lead to the two other modifications of the fibrine, may be pursuing its course. Thus we may have at the same period the tissues invaded by an inflammation which for a long period is confined to its first degree of intensity; other portions affected with greater, or, as we may call it, a second degree of intensity; and still other points, which however are for the most part very limited, in which the process goes on with its highest intensity. In such a case the blood will furnish a very consistent coagulum, and one rich in fibrine, which will cover it with a thick coriaceous buff, and upon the surface of this gelatiniform deposits will be observed." P. 371.

The reasons which have induced the author to consider the *parafibrine or gelatiniform crust* to denote the highest intensity of the inflammatory process are the following. 1. It appears much more frequently in the earlier than in the later periods of the disease, when all the symptoms denote the activity of the process; and disappears before the *bradifibrine* (or *coriaceous crust*), and long before the simply increased fibrine (*firm coagulum*) as the disease advances towards its cure. 2. It appears as a consequence of intense inflammation of the skin excited by blisters, &c., but not when this exists in a milder degree. 3. It is found in the fluids produced in various intense inflammations, such as pleurisy, &c. In a note the author states, that Dr. Gamberini, one of the most careful observers in Italy, has informed him that he hardly recollects a case in which the appearance of gelatiniform flakes and lines upon the clot was not accompanied with great intensity of the disease.

### Series IV.—On the Effects of the Abstraction of Blood upon the Human Organism.

"If the abstraction of blood were confined to the production of a greater or less diminution of this fluid, and to a proportionate decrease in the vital power and in the functions of the part which most nearly depend upon it, the question of bloodletting would be one of such simplicity that its mere proposition would suffice for its solution. But the effect of bleeding cannot be regarded as a direct diminution of vital power solely because it induces a direct diminution of the material for the exercise of life; nor as an enfeeblement of that power because it directly lessens a means necessary for its manifestation. It may and does produce these effects, not however in a direct way, but by a series of successive and reciprocal modifications, that furnish an explanation of all the various phenomena, to which the loss of blood, whether in the physiological or morbid condition, gives rise to.

"*The first modification which the abstraction of blood induces in the organism is exerted upon the blood itself;* a modification both immediate and great, though hitherto not noticed, or only superficially so, by clinical observers. And it is from this modification that all the other multiplied phenomena originate. It is from having commenced the examination of the different effects of bloodletting in an inverse order—from having sought for the material and functional changes in the solids consequent upon it, before examining what changes the circulating residuum itself had undergone—that the study of this subject has proved arduous, vague, and, I may add, of little utility. I have no intention to attempt the examination of the intimate dynamic or molecular changes which the blood under this influence undergoes, because I do not feel certain of the utility of inquiries during which the senses do not continuously bear us company; nor have I the intention of submitting the blood to chemical decomposition and the forced isolation of its principles by means of re-agents and analyses, because the indications derivable from these do not seem to me all equally to be relied upon. But I believe that the mere accurate determination of the principal sensible phenomena which the blood under such circumstances presents, may be so conducted as to produce an assemblage of precise expressions of its manner of modifying itself in strict relation with its material conditions, and the amount of vitality it indicates. And it is the result of the study of such modifications which I am about briefly to advert to, with above all the intention of showing the extent to which we may determine with exactitude the *influence of bloodletting on the cure of diseases;* and of what importance the determination of this is in clinical practice."—Vol. 121, p. 7.

The mass of the blood may be modified after venesection as respects its *quantity,* its *quality,* and its *motion.*

1. *Effects of Bloodletting upon the Quantity of the Blood.*—It is an error to suppose that the mass of the blood becomes diminished in quantity in proportion to the amount abstracted. To prove this, it is only necessary to examine, by means of the areometer, portions taken at the commencement and at the conclusion of a venesection, when the latter will be almost invariably found to be of less density than the former. M. Polli has supplied several tables deduced from often-repeated investigations of the exact amount of this diminution of density, both during any one bleeding, or from one bleeding to another, when these have been repeated. The following are the conclusions at which he arrives.

" 1. The blood which at the end of the emission possesses a less density than that first drawn, and its serum which is often found to be of greater density than that which accompanies the first blood (though of lesser than the whole mass of the blood), prove that, during the very act of bleeding, there is absorbed into the circulating channels a greater or less quantity of serum of varying density. 2. The quantity of serum that enters the circulation during an ordinary bloodletting, to induce in the totality of the remaining mass the mean dilution which the areometer exhibits, is approximatively equivalent to a thirtieth part by weight of the remaining blood. 3. The quantity of serum which is absorbed from one bleeding to another in an interval of twelve hours, is about double that amount. 4. The dilution of the blood which takes place during bleeding is principally produced by the mechanical forcing into the vessels, by means of atmospheric pressure, of the fluids which everywhere moisten the tissues; but the dilution which takes place from one bleeding to another, is the effect of an absorption governed by the vital laws." P. 27.

There are certain facts, also, which are explained by the above circumstances, such as the thirst which patients so often experience after bleeding; the dryness of the mouth and fauces, which women suffering from great metrorrhagia complain of; the diminution or even complete absorption of some dropsies after free bleeding, &c.

" As regards the question before us, the human body may be regarded as a spongy tissue, entirely permeable by the different humours which fill its cells and freely traverse its vessels. The atmospheric pressure to which it is submitted not allowing that any cell or any vessel shall remain empty or only partially filled, is continually propelling the liquids to whence least resistance offers itself, co-operating with the vital action of the tissues and the fibres which constantly contract on the volume of the contained fluids. During the aspiration which is immediately produced by opening a vein, the liquids which proceed constantly to dilute the mass of the blood may be regarded then as only mechanically driven into the vessels, and they do not in fact exceed in quantity the amount of blood lost; but, in the intervals which occur between one bloodletting and another, the diluting liquids are physiologically introduced by means of the absorbents, when too they are more elaborated, and often temporarily in greater quantity than suffices to replace the blood taken. And this explanation leads to the establishment of the difference which exists between a free evacuation of blood at one time, and a number of smaller abstractions made at different periods, although, taken altogether, not more blood is taken by the one plan than by the other." P. 23.

*Practical Applications.*—The fact of the rapid absorption of fluids which supervenes on venesection may be advantageously borne in mind when we have in view the augmentation of the operation of certain medicinal substances, the diminution of the density of the blood, and the removal of effusions from important organs; while, when we wish to delay absorption in the case of poisoning, purulent infection, &c., we must abstain from the abstraction of blood. Employed during the existence of an abscess, and especially if this is in communication with the air, it would become but the means of inducing a more general poisoning of the blood. So, just as the operation of purgatives and emetics long taken without any effect being produced is thus much expedited; and a prudent emission of blood will favour the specific action of mercury; so in recent poisoning, and in those somewhat similar cases of gastric-bilious saburræ of an acrid and putrefective character, venesection but hastens the fatal result. "To the same cause, also, we may attribute the promptly fatal termination of certain chronic affections of spoiled organs, which nature had nevertheless endeavoured to maintain in an isolated condition, and which uncompromised by bleeding might not so readily have terminated life."

2. *Effects of Bloodletting upon the Qualities of the Blood.*—The blood may be *qualitatively* modified in respect to its *density*, its *coagulability*, and its *temperature*.

(A). *Modifications of the Density of the Blood.*—These have been already briefly alluded to in the foregoing section: but the author here enters into the subject in great detail, exhibiting the tabular results of observations he has made on patients, and of numerous experiments he has performed on the horse. The questions he announces for solution are—1. What are the laws which govern the diminution of the density of the blood in general, during the same bleeding, and in the intervals of different bleedings? 2. What are the laws which govern the recovery of its density by the blood, after the cessation of such evacuations? 3. What is the relation which these modifications of the entire blood bear to those of its serum; and to what extent do they also take place in respect to its other component parts? To follow him through the detailed replies he furnishes, would far exceed our limits; but we may state, as the general result, that the density of the blood was found to diminish in proportion to the amount abstracted; and to be recovered after the abstraction had ceased, in proportion as the individual had become otherwise restored to his normal condition. Comparing the condition of the mass of the blood with that of its serum, "the blood loses nearly $2\frac{1}{2}$ of its cruor to one of the solid materials of the serum, as the effect of several bleedings; while, during the same bloodletting, the loss seems exclusively to affect the cruor, the serum almost always increasing in density." The relative condition of the various component parts of the blood is thus stated :—

"The speedy dilution of this fluid is far from merely consisting in a simple watery addition to its mass; and the term *Hydræmia,* proposed to distinguish this particular condition, would only express one of the modifications it undergoes, and that perhaps the least important. Recapitulating the variations which the blood presents in its principles, with a diminution of its density, and availing ourselves not only of our own areometrical data, but also of the analytical results declared by hæmatologists in recent times, we may conclude

"1. That, as a consequence of a *series of moderate bloodlettings* practised at short intervals in the same individual, the *fibrine* is stationary; the *red globules* and *albumen* diminished; the *saline, fatty,* and *extractive* matters are rather stationary than in excess; and the *water* is increased. 2. That, at the conclusion of a single *very free bloodletting,* the *fibrine* is increased; the *red globules* are diminished; the *albumen* is stationary or increased; the *saline, fatty,* and *extractive matters,* and the *water* are increased. 3. That, as a more remote consequence of *one very abundant blood-letting,* the *fibrine* diminishes; the *red globules* continue to diminish; the albumen is diminished; the *saline matters, extractive, fatty matters* and *water increase.*

"The materials most easily reproduced in the circulatory mass after abstraction are then the water, the saline and extractive matters, and the fibrine; the albumen is reproduced with more difficulty, but the red globules with much more so. One of the effects of bloodletting the least easy to remove is the *defect of red globules;* and as these are the most vital portion of the fluid, constituting almost organized beings functioning of themselves, we may say that blood-letting tends to deprive the blood of its most characteristic element: and this condition of the fluid will consequently be more faithfully expressed by the term *despoliation* (*spogliazione*), employed a century ago by Quesnay, than by the terms *hydræmia* or *anæmia,* used by the moderns.

"By bleeding, then, the blood is despoiled of its most noble constituents, becomes watery, and is mixed with ill-elaborated materials, having little affinity with it. The vital excitement of the tissues and nerves is thus intercepted and depressed, just as it would be by a direct injection of foreign matters into the blood; so that, in this point of view, bloodletting is a species of poisoning of the blood itself, greater or less according to the quantity taken and the mode of its abstraction. And it is of this poisoning of the blood, as well as of the deprivation of its more perfect constituents, that medicine avails itself, when abstracting blood, to overcome or moderate processes of irritation or inflammation; it presents directly to the patient thus suffering, as succedaneous and adjuvatory to blood-letting, small doses of poison, which absorbed into the blood, reproduce in part the good effects directly derivable from blood-letting."—Vol. 121, p. 79,

An increased *quantity of fibrine* after repeated bloodletting is not only found in man suffering under inflammation, and kept on low diet; but also in healthy horses kept also on short commons, and bled day by day. The evacuation of blood alone, then, leads to the production of fibrine as noticed already by Pessina and others.

"In our experiments upon the horse we have stated that the thickness of the crust was proportioned to the frequency of the abstraction; so that when, after the first bleeding, it formed but a third or a fourth of the coagulum, after the last it constituted four-fifths. The principal cause of this increasing thickness of the crust is the diminished density of the blood and the paucity of red corpuscles, by reason of which these are enabled speedily to descend to the bottom of the recipient, where they occupy a small space only, leaving a proportionally larger stratum to figure as a crust. This crust may be called the *false buff* or *buffiness from bloodletting,* to distinguish it from the *phlogistic buffiness,* which occurs in blood far denser and more rich in red globules, and whose principal cause is slowness of coagulation." P. 72.

(B). *Modifications of the Coagulability of the Blood.*—This point has been too minutely considered in our abstract of the First Series of the author's Researches to require further notice here. We may merely observe that his experiments upon horses confirmed the conclusions he arrived at at the bed-side, and which he sums up in the following proposition. "*In proportion to the quantity of blood taken at one bloodletting, the shortness of the interval which transpires between different bleedings, and to the number of these abstractions practised on the same individual, so much the more readily will the blood be found to coagulate, or so much the less resistance will it present to the coagulation of its fibrine.*"

(C). *Modification of the Temperature of the Blood.*—The investigations of Nasse, Marshall Hall, and Popp, are adverted to in this section : and from these, together with a numerous series of original observations, Dr. Polli arrives at the following conclusion :

"That the *immediate effect* of bleeding is to *diminish the temperature* of the remaining blood, and of the subject to which it belongs; but that, *some time after the bleeding,* and upon the *repetition of the bleeding* in the same individual, an opposite effect, or an *elevation of the temperature,* is produced.

"The causes, by reason of which among the effects consequent on bloodletting there is an elevation of temperature, are complex, and it must suffice here to indicate them. 1. The acceleration of the circulation, which renders the respiration also more frequent, and the pulmonary hæmatosis more active. 2. The introduction of combustible substances, especially fatty matters, by means of the more active absorption induced by the bloodletting, the which, when commingled with the inspired oxygen, must necessarily furnish new elements of calorification."—Vol. 121, p. 267.

(D). *Practical Applications.*—(1) In the inflammatory diseases of the pulmonary organs, the blood can be held in an attenuated and diluted state under the influence of bloodletting to a far greater extent than by the imbibition of aqueous fluids; and in this way is expectoration rendered easier than it otherwise would be.

(2) Profuse sweating, abundant alvine excretions, prolonged abstinence from drink, and whatever increases pulmonary exhalation (as exercise, a warm, dry, or rarified air), render the blood more dense ; and hence some of the benefit which persons of lymphatic constitutions derive from a residence in mountainous regions, and the free use of purgatives. Becquerel has pointed out the fact of the frequent use of *purgatives* rendering the urine scarce and sedimentiferous : and, as a strict relation prevails between the density of the urine and that of the blood, care should be taken, in the case of *pure plethora,* not to aggravate, by the use of these substances, a condition of the blood which is only relievable by bloodletting.

(3) It is not a matter of indifference whether a *given amount of blood be abstracted at one bleeding or at several.* By the first plan a much more complete and sudden modification of its mass is produced, and an effect to the same amount would require three times as much blood to be taken by small bleedings for its production: giving rise to a greater loss of red globules, a slower reparation, and a more tedious convalescence. The smaller bleedings allow of an imperfect reparation in the intervals, but the greater tolerance thus induced is a deceptive phenomenon when full bleeding is indicated, although, under other circumstances, it may be made usefully available.

"Free abstraction is indicated when we have to do with a commencing inflammation of a severe character in a robust individual; while the smaller loss is indicated in feeble subjects, liable to disturbances of the circulation, or endowed with great nervous susceptibility. In case of collections of corrupt matters (as abscess, open cancer, gangrenous foci, &c.), a large abstraction is never advisable; while in *marked plethora* (dense blood too rich in globules) it is indicated. Free abstraction, developing in great part also a mechanical effect, is required in those hydraulic derangements of the circulation arising from remora, or partial fulness of vessels; and in cases in which it is important to excite, at whatever cost, a rapid absorption of effused fluids. Small, interrupted, emissions, will, on the other hand, be always preferable, when, in a diseased state of the circulatory organs, rapid changes of the fluid in them, or a difficult assimilation of fluids absorbed without much election, is to be feared."—Vol. 121, p. 281.

(4). The phenomena observed in the excessive loss of blood in *hæmorrhages* teaches us two things. *First.* That a profuse loss of blood is best borne when taken so as to imitate in some degree the stillicido of hæmorrhage than if abstracted rapidly by a free bleeding. Quesnay showed that, if a large opening be made in a vein, we can regulate the flow by means of a bandage just as we please; and, if it be so managed that about six ounces only are lost in the hour, the patient will be found able to tolerate such an evacuation for a very long period; so that at the end of the 24 hours, perhaps, 12lbs. (of 12 ozs.) may have been taken.

"In suffocative angina, in grave pulmonary congestions threatening asphyxia, in certain very rapid cerebral phlegmasiæ, and, above all, in cases in which energetic and prompt treatment is demanded, while the tendency of the patient to syncope impedes its adoption, or gives rise to functional disturbances it is desired to avoid, this means of bloodletting becomes of great use."

*Secondly.* When we are desirous of *arresting a hæmorrhage*, we may often accomplish this by drawing a small quantity of blood in a large stream; so that, with no great loss and endured for a very short period, coagulation at the mouths of the affected vessels may be promoted. This prevents that despoiling of the blood and exhaustion of the strength which supervene on a constant drain of blood, surprisingly as the system may seem to tolerate this.

"And it is not unreasonable to suppose that many cases of metrorrhagia, hæmoptysis, epistaxis, and even hæmorrhage from mechanical lesions of internal vessels, to combat which a number of interrupted bleedings have been in vain employed, besides a variety of cold and hæmostatic applications, would have been easily checked, had the patient been boldly bled, *ad deliquium*, at the commencement."

(5). It is a well-established fact, that excessive bloodletting, and even moderate in subjects having diseased circulating organs, give rise to *dropsical effusions*. This does not seem to be dependent upon a disturbed state of the circulation, or on any venous stasis, but may be, in our author's view, referred to three causes. (*a*). In such persons there is induced in the remaining mass of the blood the same serious modifications observable in Bright's disease—watery dilution, diminution of globules, and great impoverishment of albumen. (*b*). After repeated bleeding, the secretory organs become inert and indolent. (*c*). The last cause, probably the foundation of the other two, is found in the diminished density of the blood, allowing it to yield more readily to the capillary attraction of the membranes, and thus give rise to an exosmosis or hydropic effusion which would not occur in denser blood.

This leads us to an appreciation of the *advantageous employment* of *blisters* for the cure of dropsies dependent upon a modification of the crasis of the blood. By free vesication we may take from a patient from 8 to 16 ozs. of a fluid which is a species of diluted blood—" thus evacuating all the materials of the blood except the red globules, practising, in fact, a *white bloodletting*, acting upon the remaining mass in a completely opposite manner to its spoliation by bleeding. This is probably the chief reason for the great assistance all practitioners have derived from the use of blisters at the close of the treatment of inflammation, when much blood has been abstracted, and the removal of a commencing effusion is desired. How can we in such cases better remedy both the effects of the disease and the treatment than by the use of blisters ?" Large, flying blisters are best adapted to this end.

(6). The attenuation of the blood by repeated bleeding, its deprivation of red particles, and the excess of fibrine, induce an amount of crust or buffiness which would not result from the mere slow coagulation of phlogosis. A *false buff, or a buffiness from bleeding,* may thus become very marked, and a practitioner ignorant of its mode of generation may draw very unsafe indications from its inspection.

(7). The question whether bleeding, used immoderately, or in cases in which it is not strictly necessary, may not *give rise to a necessity for its more frequent repetition,* a habit, is answered affirmatively for the following reasons.

" (*a*). That it accustoms the organism to tolerate a change in the composition of the mass of the blood which renders the effects of subsequent abstractions less sensible. (*b*). It habituates it to the renunciation of natural modes of restoration, or such as, by means of a little expectation, might easily be made to appear: so that after one, two, or three morbid emergencies, cured in this oblique manner, on a fourth occurring, bleeding becomes inevitable. (*c*.) A reparation of blood greater than the loss is sometimes provoked, giving rise to a species of *false plethora*."

(8). Not only may excessive bleeding render convalescence tedious, and give rise to cachexiæ and vices of innervation difficult of removal, but it may lead to a fatal termination of the malady, which would not otherwise have taken place. The effects of repeated bleeding are accumulative ; and the apparent toleration exhibited by the individual in so few signs being offered of disturbed functions is a far less faithful guide than is the examination of the changes operated in the blood. These are progressive, as the fluid cannot be re-constituted when the abstractions succeed each other at short intervals. The effect does not terminate with the bleeding ; for the absorption of new materials, and the influence of these upon the blood, continue long after.

To the remaining section on the "*Effects of Bloodletting upon the Motion of the Blood*," we have space only to allude. The experiments of De Heide, Haller, and Spallanzani upon cold-blooded animals, together with his own on horses, and his observation of man suffering under disease, lead the author to the general conclusions that the *immediate* effect of bloodletting both on man and animals is the production of a *greater quickness* of the pulse: but, in both, when carried to *syncopy*, it *diminishes* the number of beats. *Repeated abstraction* not only tends to *accelerate the circulation*, but also, in a still greater degree, the *respiration;* and this increased frequency of pulse and respiration are among the effects of bloodletting latest in disappearing.

This brings Dr. Polli's valuable labours to a conclusion, as terminating the investigation of the *immediate modifications* which the blood undergoes. To complete the subject, the effects which the fluid so modified produces on the various organs has yet to be examined; and we hope this able enquirer will not recoil before a task he declares too difficult and laborious for him to encounter. Certain are we that a more careful and talented investigator will not be found; and in no country is such an enquiry more urgently called for than in Italy, where it seems to us that this therapeutical agent is employed with a most unjustifiable freedom and rashness.

